# Current Treatment for Myositis

**DOI:** 10.1007/s40674-018-0106-2

**Published:** 2018-09-15

**Authors:** Simone Barsotti, Ingrid E. Lundberg

**Affiliations:** 1 Rheumatology Unit, Department of Clinical and Experimental Medicine, University of Pisa, Pisa, Italy; 2 Department of Medical Biotechnology, University of Siena, Siena, Italy; 3 Division of Rheumatology, Department of Medicine, Karolinska Institutet and Karolinska University Hospital, Solna, SE-171 76 Stockholm, Sweden

**Keywords:** Myositis, Idiopathic inflammatory myopathies, Treatment, Immunosuppressant, Clinical phenotype

## Abstract

**Purpose of review:**

The purpose of this review was to give an update on treatment modalities for patients with idiopathic inflammatory myopathies, or shortly myositis, excluding the subgroup inclusion body myositis, based on a literature survey on therapies used in myositis. Few controlled trials have been performed in patients with myositis; therefore, we also included a summary of open-label trials, case series, and case reports.

**Recent findings:**

Glucocorticoid (GC) in high doses is still the first-line treatment of patients with myositis. There is a general recommendation to combine GCs with another immunosuppressive agent in the early phase of disease to better control disease activity and possibly to reduce the risk for GC-related side effects. Furthermore, combining pharmacological treatment with individualized and supervised exercise can be recommended based on evidence. There is some evidence for the effect of rituximab in patients with certain myositis-specific autoantibodies, whereas other biologic agents are currently being tested in clinical trials.

**Summary:**

Immunosuppressive treatment in combination with exercise is recommended for patients with myositis to reduce disease activity and improve muscle performance. Subgrouping of patients into clinical and serological subtypes may be a way to identify biomarkers for response to specific immunosuppressive and biological agents and should be considered in future trials.

## Introduction

Idiopathic inflammatory myopathies (IIMs) are a heterogeneous group of autoimmune diseases, mainly characterized by inflammation of the skeletal muscles, but involvement of internal organs such as lungs, heart, and esophagus is common. Traditionally, IIMs are classifiable in three subtypes, polymyositis (PM), dermatomyositis (DM), and inclusion body myositis (IBM), but recently, also other subgroups, such as necrotizing autoimmune myopathy (NAM) and anti-synthetase syndrome have been identified [1•].

There are no standardized therapeutic guidelines for treatment of IIM, particularly due to the lack of randomized controlled trials and due to the rarity of the disease. Furthermore, the presence of different disease subtypes makes it difficult to design clinical trials. Therefore, the therapeutic approach is mainly guided by expert opinion and case series.

In order to improve the standardization of the patients enrolled in clinical trials, recently, the new European League Against Rheumatism/American College of Rheumatology (EULAR/ACR) classification criteria for adult and juvenile idiopathic inflammatory myopathies and their major subgroups [2••] have been published. Additionally, to homogenize the assessment of the disease activity during clinical trials, EULAR/ACR criteria for minimal, moderate, and major clinical response [3] have been proposed.

Despite these proposals and several ongoing trials, glucocorticoids (GCs) remain the first-line therapy in treatment of IIM, but the use of immunosuppressive drugs as adjunctive therapy is increasing in the early phases of the disease, both for adjunctive efficacy and for their steroid sparing effect [4]. Even if few evidence-based data are available, the immunosuppressant should preferably be chosen according to the patient’s clinical characteristics and main organ involvement (Table 1).

**Table 1:** List of drugs indicated for the treatment of IIMs and their efficacy reported in literature according to clinical/serological characteristics. The black boxes indicate the possible indication of the drugs

		GCs	MTX	AZA	CYATAC	MMF	CYC	HCQ	IVIG	RTX	Physical therapy
Disease involvement	Muscular	■	■	■	■	■	■	A	■	■	■
	Skin	■	■			■	■	■	■	■	
	ILD	■	B	■	■	■	■		■	■	
	Arthritis	■	■							■	
	Dysphagia	■							■	■	
Auto-Ab positivity	Anti-synthetase				■					■	
	Mi-2									■	
	SRP									■	

A: the patients should be monitored for worsening of myalgia and skin disease

B: the patients should be monitored for possible drug-related lung toxicity

*GCs* glucocorticoids, *MTX* methotrexate, *AZA* azathioprine, *CYA* cyclosporine, *TAC* tacrolimus, *MMF* mycophenolate mofetil, *CYC* cyclophosphamide, *HCQ* hydroxychloroquine, *IVIG* intravenous immunoglobulins, *RTX* rituximab, *ILD* interstitial lung disease

The aim of this review is to provide currently available evidence for GCs and traditional immunotherapy in the treatment of IIM and to provide an overview of the novel therapies available for treatment of refractory patients. As most patients with IBM are refractory to immunosuppressive treatment, our focus in this review is the other subtypes of IIM.

## Treatment

### Pharmacological treatment

#### Glucocorticoids

Despite the absence of clear data derived from randomized controlled trials, treatment with GCs remains the first therapeutic approach in clinical practice for patients with IIMs [4]. The treatment is able to reduce muscular inflammation [5], and more than 60% of the patients show improvement of muscle symptoms when treated with GCs [6]. This occurs in particular in the first 6 months after the start of the treatment [7]. High GC doses have also been used successfully for lung involvement [8].

In the traditional therapeutic approach, GCs were started with 1 mg/kg/day of prednisone or equivalent [9]. Lower dosage should be considered in the case of mild disease or, when contraindications are present, such as diabetes mellitus, hypertension, or glaucoma [4], and higher dosage, up to 2 mg/kg/day, can be prescribed in selected cases but not exceeding 80–100 mg/day [10]. In patients with severe disease, such as those with severe muscle impairment, dysphagia, rapidly progressive interstitial lung disease (ILD), or skin ulcers [11], an induction therapy with intravenous methylprednisolone pulses (500–1000 mg/day for three consecutive days) is often recommended [12].

Different schemes for GC tapering have been proposed but usually, the higher dose should be maintained for 2–4 weeks then gradually reduced by 20–25% each month until 5–10 mg/day of prednisone or equivalent dose is reached [4, 13]. During the reduction of GCs, a strict clinical and laboratory follow-up should be performed with monitoring of muscular strength with validated methods (e.g., manual muscle test 8 (MMT8)) and serum muscular enzymes [4, 10].

Side effects (SEs) are common in patients treated with GCs [14] and include diabetes mellitus, systemic artery hypertension, dyslipidemia, osteoporosis, weight gain, cushingoid appearance, skin thinning, gastric intolerance, mood changes, infections, hirsutism, cataract, and glaucoma [15]. Some studies suggest that dexamethasone oral pulses as induction therapy may have similar efficacy as methylprednisolone, but with fewer side effects [16].

Although GCs are usually effective in the treatment of IIMs, at least to some extent, a great number of patients require the addition of another immunosuppressive drug for refractory disease [6, 17], disease flares, and skin involvement or to reduce the GC cumulative dose. In each patient with a recent-onset IIM, the prescription of an immunosuppressive agent should be considered from the first phase of treatment, as early treatment is associated with a better outcome [6].

#### Adrenocorticotropic hormone gel

The effects of adrenocorticotropic hormone gel (ACTH gel) were studied in a retrospective case series [18] and in a recent open-label trial [19•]. ACTH gel was approved already in 1952 by the US food and drug administration (FDA) for the treatment of IIMs but has not been approved by the European Medicines Agency (EMA). The treatment improved muscular strength and skin rash, with an average improvement of 19.3% of the muscular strength measured by MMT in 71% of patients, reduction of cutaneous VAS of 88% in 4/5 patients with dermatomyositis skin rash and allowing a reduction of the GC dosage [18, 19•]. ACTH gel was administered subcutaneously once or twice a week for 12 weeks. Despite that the therapy was well tolerated without major side effects, further studies are needed to evaluate the safety and efficacy of ACTH gel in patients with IIM.

#### Hydroxychloroquine

As reported in observational studies, treatment of skin involvement in DM with antimalarials (hydroxychloroquine (HCQ) or quinacrine) in combination with GCs with or without immunosuppressors is effective in 40–75% of the patients [20, 21] and it has been included in a recent consensus for the treatment of rash in juvenile DM [22]. Antimalarials seem to be ineffective on muscular involvement [20, 21]. The most prescribed antimalarial in routinely clinical practice is HCQ up to 400 mg/day.

The treatment is usually well tolerated but adverse events, albeit mild, are relatively common with skin eruption, gastrointestinal toxicity (nausea, vomiting, diarrhea), dizziness, and headache [23]. Antimalarial retinopathy represents the most severe adverse event; thus, there is a recommendation by the American Academy of Ophthalmology that patients should be screened at the baseline with a fundus oculi examination and during the treatment a periodical evaluation with automated visual fields and/or optical coherence tomography should be performed every 5 years [24]. Although the prevalence was unknown, a myopathy (myalgias with increase of muscular enzymes or a myopathy with vacuoles) may occasionally occur during the treatment [25], while a paradoxical worsening of the skin lesions has been reported in up to 30% of DM patients during the treatment [26].

#### Methotrexate

Methotrexate (MTX), together with azathioprine, is considered as the first choice of immunotherapeutic agents to treat the muscular involvement in IIM [4, 10]. Although no placebo-controlled trials have been published, the efficacy of MTX has been investigated in open-label studies [27] and in comparison with other immunosuppressants [28–30]. MTX has also been successfully used in the treatment of skin manifestations of DM patients [31, 32] although with conflicting results [33].

A randomized, open-label trial to assess the efficacy and safety of MTX combined with GCs vs. GCs alone is ongoing [34]. MTX can be administered orally, subcutaneously, or intramuscularly up to 20–25 mg/weeks. MTX administration should be followed by an adequate administration of folic/folinic acid to reduce side effects and patient withdrawal from MTX [35]. The use of MTX during pregnancy is contraindicated in women because of teratogenic effects [36].

The most common adverse event (AE) of MTX are infections, hepatotoxicity (liver enzyme elevation and cirrhosis), and blood cell count alterations and transaminases and complete blood cell count should be routinely tested in patients treated with MTX [36]. MTX should be carefully used in people with impaired renal function and in patients also treated with nonsteroidal anti-inflammatory drugs. Pulmonary toxicity in patients treated with MTX represents a serious and unpredictable side effect [37]; thus, other immunosuppressive drugs are usually recommended as first-line therapy in combination with GCs in patients with IIM related ILD [38].

#### Azathioprine

Azathioprine (AZA) is a purine analogue and acts as antimetabolite, blocking the purine and metabolism and the RNA and DNA synthesis. The GC sparing effect of AZA was investigated in a randomized placebo-controlled trial in patients with PM. Although the combination therapy was not superior to GC alone after 12 weeks, after 3 years extension, the combination of GC with AZA allowed reduction of the prednisone daily dose and was associated with a better functional outcome [39, 40]. Even if the certainty of the evidence is very low, AZA has also demonstrated efficacy in retrospective case series for the treatment of IIM-ILD [41] and could be considered in the treatment of patients at high risk to develop MTX-associated lung injury. Even if no head to head trial has been conducted, AZA has a similar efficacy compared to MTX [6, 42], and also, the survival in patients treated with AZA seems to be similar or better [43] compared to patients treated with MTX although confounding by indication cannot be excluded.

AZA is usually administered orally starting with 50 mg/day and with subsequent incremental increase by 25–50 mg every 1–2 weeks up to 2 mg/kg/day [4, 10]. Complete blood cell count, liver enzymes, and kidney function should be routinely tested in patients receiving AZA.

Common SEs include vomiting, liver toxicity, and bone marrow suppression [44]. The latter SE is particularly common in people with a genetic deficiency of the enzyme thiopurine S-methyltransferase that can be genotyped before AZA prescription [44]. Although AZA is officially contraindicated during pregnancy, substantial data support the safety of the drug during pregnancy [45]. Among the drug interactions, particular attention should be given in avoiding concomitant treatment with allopurinol [44].

In patients who failed to respond to MTX or AZA alone, the combination of these two agents can be considered, and up to 53% of refractory patients improved their muscular strength and in capacity of performing daily activities with only minor adverse events [30].

#### Calcineurin inhibitors

Cyclosporine-A (CYA) and tacrolimus (TAC) are calcineurin inhibitors (CNIs) and their main effects are the inhibition of T cell activation and reduction of the activity of genes coding for IL-2 and related cytokines. As T cells play an important role in the pathogenesis of myositis [1] and of IIM-ILD [46], the rationale of the use of CNI in IIM patients is high.

In addition to the improvement of muscular involvement, the treatment with CNI has been associated with better respiratory outcome in patients with DM and PM and ILD, allowing an improvement of both the pulmonary function tests (PFTs) and high-resolution computed tomography (HRCT) [47–50], particularly if started during the early stage of the disease [51]. Anti-aminoacyl-tRNA synthetase (anti-ARS) autoantibody positivity seems to be related to a good effect of CNI [49, 52, 53]. No significant differences have been identified between CYA versus MTX for muscular involvement, when they were compared in a trial [29].

CYA is usually administered orally from 2 to 4 mg/kg/day divided in two daily doses [47]. TAC can be started initially at a dose of 1 mg twice a day titrated with 1–2 mg/day until the target blood levels of 5–20 ng/ml were reached [49]. Serum levels of both drugs should be carefully monitored to maintain the therapeutic range and to avoid severe adverse events [49, 51].

Risk of toxicity with CNI treatment includes nephrotoxicity, hepatotoxicity, hypertension, hypertrichosis, gingival hyperplasia, headache, and the risk increases with increasing doses of the drugs [54]. Serum creatinine, liver enzymes, complete cell blood count, and blood pressure should be repeatedly evaluated during the treatment especially in the first 3 months [54]. Since CYA and TAC influence the cytochrome P 3A4 (CYP3A4), awareness of potential drug interactions is important and careful checking of other medications should be done before starting treatment with CYA and TAC [54, 55]. Additionally, grapefruit juice may interfere with CYP3A4 metabolism [55].

#### Mycophenolate mofetil

Mycophenolate mofetil (MMF) is a prodrug that inhibits T and B cell proliferation reducing the guanosine nucleotide synthesis. The efficacy of MMF in IIM patients has been reported in small case series [56, 57] and an open-label study in combination with intravenous immunoglobulins [58].

MMF seems to be effective in the treatment of skin manifestation in DM patients [59]. Interestingly, a good response to MMF was also reported in IIM-ILD patients, even with rapidly progressive ILD [60]. After the treatment with MMF, an improvement of respiratory symptoms, PFTs, and diffusing capacity of the lungs for carbon monoxide (DLCO) was recorded [61, 62].

MMF is administered orally starting from 500 mg twice a day up to 2–3 g daily dose refracted in two daily administrations [4]. MMF is commonly well tolerated but gastrointestinal symptoms (nausea, vomiting, and diarrhea) and blood cell count abnormalities, including severe neutropenia, may occur. Patients should be monitored with complete blood count, liver enzymes, and renal function. Higher susceptibility to opportunistic infections, also severe, has been reported [57]. The treatment with MMF is not recommended during pregnancy [45].

#### Cyclophosphamide

Developed as antineoplastic chemotherapy, cyclophosphamide (CYC) has been used to treat severe manifestations of rheumatic diseases, in particular in patients with progressive ILD. In IIM patients, the use of CYC is reported in anecdotal case series. The use of this drug has been mainly reserved for patients with severe or progressive ILD and it has been reported to reduce respiratory symptoms, allowing the improvement of chest HRCT and improvement of PFTs [63–65]. Combination therapy between CYA and CYC may be a promising treatment for rapidly progressive ILD in DM [66], even if the results can be unsatisfactory for some patients [67]. Also, combination therapy with rituximab has been reported to be useful in patients with severe ILD with anti-MDA5 autoantibodies [68]. Even if usually reserved to the treatment of lung involvement, CYC has been reported to be effective also on muscular symptoms, allowing improvement of muscular strength and reduction of serum levels of muscular enzymes [65].

Different treatment protocols have been reported in the medical literature with no clear advantage of one therapeutic scheme compared to the others and the optimal dose and duration is not yet defined. The most frequently used therapeutic approach was with IV pulses (0.3–1 g/m^2^ or 10–30 mg/kg applied at weekly to monthly intervals for 6–12 months) [65] but also oral administration can be prescribed [69]. Recently, a lower dosage approach as in lupus nephritis has been proposed (500 mg every other week up to 12 administrations) with good efficacy and without major adverse events [70].

The treatment with CYC is limited by potentially severe adverse events, such as myelosuppression (neutropenia), hepatotoxicity, renal toxicity, hemorrhagic cystitis, infections, irreversible ovarian failure leading to infertility, and secondary malignancy [71]. The side effects on kidneys and the urinary tract can be reduced with aggressive hydration with forced diuresis and sodium 2-mercaptoethanesulfonate (MESNA) premedication. Close monitoring of blood cell count and liver and renal function is mandatory during the treatment. CYC is contraindicated during pregnancy [45].

#### Intravenous immunoglobulins

Intravenous immunoglobulins (IVIG) are preparations produced from pooled IgG preparations from thousands of donors and contain antibodies directed against a broad range of pathogens, as well as against numerous foreign and self-antigens [72]. Despite that the mechanism of action of the treatment is not yet known, it probably involves different disease-specific pathways involved in the production and maintaining of inflammation and autoimmune processes [72] and it is considered an immunomodulatory rather than immunosuppressive agent [4].

The treatment has been reported to be effective in two small double-blind, controlled trials in patients with DM [73, 74] and in prospective open-label trials [58, 75, 76]. IVIG has shown effect on muscle performance involvement and skin involvement in DM [77] and has been tested also in the treatment of ILD in refractory patients [78, 79]. Dysphagia may respond to treatment with IVIG [80], also in patients with IBM, who are otherwise treatment-resistant [81, 82]. There are also negative reports on the effect of IVIG in patients with IIM [83••], as in one study which included repeated muscle biopsies, there was no significant beneficial effect on muscle performance nor on inflammation in muscle tissue [84]. Unfortunately, we lack biomarkers that may predict response to high-dose IVIG treatment, but patients with anti-HMGCR antibodies may be such a subgroup [85•].

As first proposed by Imbach et al., the therapeutic dose of IVIG was empirically fixed to 2 g/kg/months in three to five refracted administrations [86]. The number of therapeutic cycles may be variable according to the disease severity/response to the treatment. IVIG may be combined also with immunosuppressive drugs [58, 87]. Recently, subcutaneous immunoglobulin (SCIG), weekly self-administered by a programmable pump, has been reported to be a valuable alternative to IVIG in myositis. This treatment appears to be a safe and efficient alternative to hospital-based IVIG with less reduction of the patients’ quality of life [88, 89•].

IVIG is considered a relatively safe therapy. The adverse events are reported in 5–15% of the infusions and in most cases are mild and transient with headaches, fevers, chills, and myalgias. The use of high-dose IVIG in IIM patients has been anecdotally associated with congestive heart failure, hyper viscosity syndrome, hemolytic anemia, and transfusion related acute lung injury. Anaphylaxis has been reported in patients with IgA deficiency. IVIG may represent a potential risk for blood-borne transmission of undetectable infections [83••]. IVIG is considered safe in patients with infections [10] and neoplasia [90], where immunosuppressive approach may be dangerous. Although few literature data are available, IVIG can be used during pregnancy [83••]. The main limitation of the use of IVIG is their extremely high cost that allows their use mainly in severe/refractory selected patients.

### Biologics

#### Rituximab

Rituximab (RTX) is a chimeric monoclonal antibody against the protein CD20, primarily expressed on the surface of the B cells. Since 2005, several case reports, case series, open-label trials [91, 92], and reports from registries [93] have suggested a positive effect of treatment with RTX in IIM patients. More recently, one randomized controlled trial has been published [94]. Until now, data from more than 450 refractory IIM patients treated with RTX have been published with an average response rate of 78.3% [95].

The large RCT, the Rituximab in Myositis (RIM) trial, including both juvenile-onset and adult cases failed to achieve the primary efficacy endpoint [94]. However, RTX was able to reduce the clinical activity and showed an important steroid sparing effect. In a post hoc analysis, patients with anti-Jo1 or anti-Mi2 antibodies achieved a significant improvement [96]. In an open trial, conducted in 12 patients with anti-ARS antibodies positivity, RTX was effective on lung and muscular involvement [97]. RTX has been identified as the most commonly used biologic in the treatment of IIM [98•]. The main indications for RTX treatment in IIM include refractory muscular, lung [97, 99], skin [100•], or joint [10] involvement. Patients with myositis-specific autoantibodies (MSA) seem to have a better response to the treatment, in particular those with anti-ARS positivity [96, 97, 99], anti-SRP [94, 101], and Mi-2 antibodies [96]. The anti-Jo1 and anti-Mi2 autoantibody levels decreased after B cell depletion and correlated with the reduction in disease activity [102].

RTX is usually prescribed with two 1-g infusions 2 weeks apart and may be repeated after 6 months. Other therapeutic regimens proposed were 375 mg/m^2^ weekly for four consecutive weeks. The clinical meaning of periodical monitoring of peripheral CD-20 positive B cells has not yet been clarified.

Rituximab is generally well tolerated but AE are relatively frequent. Infusion reactions, also severe, are the most common AE related to RTX treatment; they occur mainly during the first infusion and their frequency decreased in patients who were premedicated with intravenous GCs, antipyretic and antihistamine drugs [103]. Due to a possible cardiotoxic effect, cardiac monitoring is recommended during and after RTX infusions in patients with a history of ischemic heart disease [103]. Infections during the treatment are relatively common, including viral and bacterial infections [95] and before treatments, pneumococcal and influenza vaccinations are recommended [104].

#### Other biologics

In the last years, different biotechnological drugs have been tested in the treatment of IIM but the number of patients treated is scarce and their use can be suggested only whenever other treatments are ineffective.

Abatacept, a fusion protein that interferes with the immune activity of T cells, has been effectively used in sporadic case reports [105].Recently, a small randomized open trial suggested a possible effect of intravenous infusions of abatacept on muscle weakness in treatment-resistant patients with PM or DM [106••]. Several trials evaluating the effect of abatacept in the treatment of IIM are ongoing [107].

Although tumor necrosis factor (TNF) may play a role in the pathogenesis of IIM, treatment of IIM patients with anti-TNFα has given controversial results. Even if some authors reported the possible role of etanercept as steroid sparing agent in DM [108], other studies did not confirm this observation and some patients worsened [109]. Despite that a recent RCT showed efficacy of infliximab (IFX) in some patients [110•], the majority of clinical data was discouraging [111, 112]. Moreover, some authors reported that anti-TNF alpha therapy may induce myositis in patients with rheumatoid arthritis, psoriatic arthritis, and psoriasis [113, 114]. In summary, TNF blockers are generally not recommended in adult patients with IIM and they should be used only when other therapies are ineffective.

As interleukin (IL)-6 has been reported to correlate with myositis disease activity, tocilizumab has been tested with promising results in two case reports [115, 116]. A trial investigating the efficacy of tocilizumab in IIM patients is ongoing (clinicaltrials.gov, NCT02043548).

Anakinra, an IL-1 receptor antagonist, was tested in case reports [117] and in a small case series [118], with positive results in some patients.

Other drugs, such as alemtuzumab [119, 120], sifalimumab [121],basiliximab [122], and tofacitinib [123] are under investigation for the treatment of IIM but their use in the routinely clinical practice is not recommended.

### Non-pharmacological treatment

#### Plasma exchange/leukapheresis

Although some reports showed encouraging results [124], plasma exchange and leukapheresis have been investigated in 39 patients in a clinical trial in 1992 without showing any significant improvement in muscle strength compared to the placebo group [125]. Apheresis treatments were related to adverse events, such as placement of a central venous catheter, major vasovagal episodes, infusion reactions, and decline in hemoglobin [125]. Thus, the use of therapeutic apheresis in DM and PM has been considered to be ineffective or harmful and the use has been discouraged by the guidelines proposed by the American Society of Apheresis [126] but it may have a role in patients with acute life-threatening, nonresponsive disease. Recently, a therapeutic protocol that included hemoperfusion with polymyxin B in addition to the standard immunosuppressive treatment has shown promising results in patients with severe ILD during the course of clinically amyopathic DM with anti-MDA5 positivity [127, 128], but controlled trials are needed to confirm this effect.

#### Physical therapy and exercise

During the last years, exercise has been identified as an important adjunct part of the treatment for patients with IIM. Exercise may improve muscular metabolism, physical capacity, autonomy, and quality of life in patients with IIM [129, 130]. Both in patients with recent-onset IIM and in patients with established disease, aerobic exercise may help in improving muscle function and quality of life without safety issues [131, 132, 133•]. The proposed exercise modalities include treadmill walking [129, 134], cycling, and also resistance training [130].

Based on accumulated data on the beneficial effect of exercise in patients with PM and DM, all patients with IIM can be recommended to start physical training as soon as possible after start of immunosuppressive treatment. To begin with, an individualized training program under the supervision of a physiotherapist is recommended. Exercise may be performed at the hospital but several studies suggest a combination of home-based training program in combination to hospital-based exercise [135]: one example of a training program was based on a 1-h exercise 3 days/week; thus, continuing exercise at home seems important to facilitate this level of intensity [136]. The beneficial effects of exercise in patients with PM and DM are more evident with medium- [137] and long-term durations [132]. Exercise load and intensity of the treatment should be individually adapted to disease activity, GC dose, and patients’ characteristics [135]. Moreover, muscle strength should be monitored on regular basis [107, 136]. Concerning the efficacy of training in IBM patients, more studies are needed [138].

#### Diet and lifestyle

Only few data about dietary intervention in IIM patients are available. In a 6-month randomized controlled trial, creatine supplement in combination with exercise was able to increase the muscular function compared to placebo in adult patients with established PM and DM with low disease activity [139], even if these data were not confirmed in patients with JDM [140]. The therapeutic regimen for adults was induction therapy with 20 g/day for 8 days, then 3 g/day as maintenance therapy. Creatine supplement was not associated with adverse events [141]; however, cautions should be taken when treating patients with renal dysfunction.

## Conclusions

A main issue in treating patients with IIM is that, due the rarity and heterogeneity of the disease, randomized controlled trials are scarce and evidence-based guidelines/recommendations are lacking. Based on available data, systemic GCs are still the first-line therapy in these patients, and immunosuppressants should be considered to be added from the first phases of the disease in order to better control the disease activity and possibly to reduce the risk for GC-related AE. Although several trials using biologics in patients with IIM are ongoing, currently rituximab is the only biologic agent that to date has demonstrated effect in some patients although a large placebo-controlled trial failed to reach its primary endpoint. Physical exercise should be started as soon as the patient is capable to do this in order to improve muscle strength and to prevent the deterioration of quality of life. Recent studies suggest that subclassification of patients, according to the clinical features and/or positivity of specific MSA, may allow us to better predict the response to a specific treatment, suggesting the possibility of tailoring the treatment to the individual patient’s characteristics.
